# Supplementary material for: Student reactions to traumatic material in literature: Implications for trigger warnings
Source: PLoS One. 2021 Mar 25;16(3):e0247579. doi: 10.1371/journal.pone.0247579 (PMC7993791; doi:10.1371/journal.pone.0247579)
Supplement: Attachment — Submitted filename: Response to Reviewers.doc [file pone.0247579.s001.doc]

**Response to Reviewers**

Thank you to the reviewers for their helpful comments. They have resulted in an improved manuscript. Below, we address each critique in turn.

One theme present in a number of critiques are questions about why we didn’t pursue more assessment or detailed information. By way of explanation, this was a multi-site study done within the context of Introductory Psychology classes at four institutions. In order for the study to be fair to the participants, it had to be kept within a 60-minute time frame. In that window, the students had to read the passage and fill out psychometrics in the initial session (~40 minutes), and then complete further questionnaires at two subsequent time points. All indications were that our participants averaged an hour to complete the study, with some students taking less time, and others longer, mostly based on how quickly they read. However, this left us limited in the number and types of questions we asked. In addition, our demographics were purposely sparse. This was done to guarantee the anonymity of the participants. For example, at one of the sites, there are only 120 students in Introductory Psychology in a given semester. Even the most basic demographics could reveal their identity to the PI or students working on the project. Given the nature of the questions we were asking about trauma and psychopathology, the benefits of collecting the additional demographics did not seem to outweigh the costs. And we went to considerable lengths. For example, in order to link participants across the three different periods, we had students each and every time answer versions of security questions which further assured their anonymity.

Despite the limitations associated with time and confidentiality, we are encouraged by the data that we collected and feel that it is a good indication of how students respond to this type of material, even if it is just an initial foray into this line of work.

**Critique:** Of particular interest is how many participants had baseline PTSD, and the extent of trauma to which participants had been exposed (not just trauma type), which can influence response. Among the participants there may be low and high cumulative trauma groups which would be worth considering in secondary analyses.

**Response:**

We have now included more information on baseline PTSD and trauma rates in our sample. For “baseline PTSD” we used the recommended PCL cut-off score of 33 during the initial assessment. The number of participants with provisional PTSD at the initial assessment was 44, resulting in a percent rate of 12% which is fairly consistent with current rates of PTSD in community samples. The rate of any type of trauma (as endorsed as “having happened to me”) was 87% for a least one of the 17 items on the LEC-5. We now have included these statistics in the text on page 8. We recognize the importance of cumulative trauma in our study outcomes but because we had not intended to assess cumulative trauma, we simply don’t have a reliable measure to do so. Our measure (LEC-5) simply asked whether a particular experience had occurred for them, but it did not evaluate the extent, severity, frequency, or longevity of the trauma. Therefore, we cannot answer the question about the “extent of trauma to which participants had been exposed”. For example, on the LEC an individual may endorse “sexual assault” had “Happened to me” but this might represent a single assault as an adult for one participant or a childhood full of sexual abuse for another. Therefore, the authors are concerned that even if we were able to generate a proxy for cumulative trauma, it might provide misleading results.

**Critique:** Outside of trauma, other factors may influence response, including personality characteristics, depression, etc, which were not assessed. It is also notable that nearly all participants with a choice chose the “triggering” narrative, but there was no assessment of desire to avoid reading passage, or questions that assessed comprehension of the passage (i.e., whether or not participants actually read it). These issues should be addressed as limitations.

**Response:**

Thank you for allowing us to clarify this issue both here and in the manuscript. As noted in our preamble, we would have liked to further assess our participants but were limited for logistical reasons. We did not get a measure of a “desire to avoid reading the passage”. Our single operational definition was whether students chose to read the alternate passage or not and we now mention this in our Limitations and suggestions for future directions. We are fairly certain, however, the participants for the most part, complied with the reading. There are a number of reasons for this. First, the initial session was in person and held in a lab or classroom with a research assistant present or readily available. There really was nothing else for the students to do during that designated time. It is possible that students did not read the passage carefully or may have skipped certain sections. We have now included further detail in the Limitations section on page 26.

**Critique:** Overall, much more detail is needed on participant characteristics for the two groups (triggering vs control passage), including trauma history, (including percentage those with trigger traumas) PTSD symptoms (including ALT-PCL at different timepoints), as well as age, income (I assume this is in the background questionnaire), and other measures collected. This should be in a table format. Information is also needed on the characteristics of the universities from which participants were sampled—urban, rural, etc. More details on procedures, including randomization, is needed. I was also curious about why only one factor of the RRPQ-R was used, and not others, particularly given their prior studies using this measure? It is unclear what the purpose of this assessment was, and what information is given with this measure vs SUDS rating. Please also report RRPQ-R Cronbach’s alpha for this study.

**Response:**

We have included all the demographics we have available in the text and in a Table, both for the participants and the study sites. We also have more detail about randomization. We used the Emotional Reactions subscale of the RRPQ-R because it is a validated subscale to assess emotional reactions to research participation. This is part of what were trying to evaluate with respect to the passage and felt it was more comprehensive than a single SUDS score. For example, we asked the following five questions:

1) The research raised emotional issues for me that I had not expected.
2) The research made me think about things I didn’t want to think about.
3) I experienced intense emotions during the research session and/or parts of the study.
4) I was emotional during the research session.
5) I gained insight about my experience through research participation.

We felt a total of these five items could tap into a broader sense of emotional reactions (empathy, anticipation, sadness, and anger) that a single measure of “distress” could not capture. And in fact, the two measures, while correlated, did dissociate in terms of study outcomes, an issue we now wrestle with in the Discussion.

We only used parts of the RRPQ-R largely due to time limitations, and partly because the other factors would have less relevance to our work. For example, the other factors on the RRPQ-R are the “Participation Factor (i.e., “I like the idea that I contribute to science”), the Personal Benefits Factor (i.e., “I found participating beneficial to me”), “Perceived Drawbacks Factor: (i.e., “I found participating boring”), and the “Global Evaluation Factor” (i.e., “I was treated with respect and dignity”). While these are all interesting questions, they really would not contribute to the aims of this study and therefore did not seem worth including. We have calculated the Cronbach’s alpha for our RRPQ-R questions in this study (alpha =0.80) and now have that included in the Method.

**Critique:** The design (analysis section) is overly vague. The authors need to be more clear in specifying a priori analysis for each of the three questions.

**Response:**

Thank you for letting us know that we were insufficient this regard. We have specified a priori hypotheses and analyses for each question on page 12.

**Critique:** All figures need more detail, including error bars, statistics in captions, symbols or other demarcation of points of statistical significance, and legends to describe each group. Colors would make it easier to differentiate the groups. Please show a figure related to Alt PCL scores at days 2 and 14.

**Response**: We have revised the figures.

**Critique:** Discussion: What do the authors make of the fact that RPPR scores were elevated in this study, but not their prior study? Similarly with SUDS being elevated post-reading in prior study but not this study?

**Response:** To clarify one point, the SUDS were elevated post reading in both studies, so we have re-written a part of the paper to minimize the confusion.

However, the emotional subscale of RRPQ-R was different between the two studies both with respect to trauma type and provisional PTSD status. In the end, this was almost certainly an issue of power and design. In the first preliminary study cited in the manuscript, it was more of a proof of concept study in which there were only 123 participants, of whom only ¼ had a triggering trauma and 18 of 123 had PCL > 33. The study was also different in design in that we manipulated the nature of the trigger warning. In one condition, we set up negative expectations, in another condition positive expectations, and in another condition neutral expectations. In addition, this study was only carried out at the two smaller, liberal arts colleges.

However, in this initial study we still found SUDS and PCL effects that we felt were worth pursuing further, which resulted in the larger study submitted for publication.

In this current manuscript, we feel that it might actually be best to remove the reference to the non-significant RRPQ-R findings in the earlier preliminary study, as that difference between the two studies is probably not meaningful and creates the kind of confusion that the reviewer articulated. We feel the findings of the current study are relatively definitive in comparison to the earlier work and supersedes the earlier findings. However, if the reviewers/editors feel that the discrepancy between the two studies is worth noting and explaining, we are happy to make the changes.

**Critique**: Alt PCL scores were not significantly higher after passage reading, but showed a strong trend (p=.07) for being higher in trigger condition, which may be an issue of statistical power given the very low sample size in the control condition. In addition, trigger trauma participants did have higher alt-PCL scores throughout although they did not report which cluster of symptoms were most highly endorsed. A symptom cluster analysis would reveal this, and these findings merit further attention in the discussion. Relatedly, if alt pcl scores were higher post-reading then the statement “The data suggest that “triggering” did not occur as a function of being exposed to the passage” is not very accurate. I think it would be more accurate to say that those with trigger traumas did in fact show a heightened trauma-related reaction to reading the passage, but this response was not sustained over time.

**Response:**

This point is well taken and we initially interpreted the statistical trend cautiously. However, the reviewer’s comments encouraged us to go back and re analyze the data both in terms of a simple effect size as well as clusters based on hyperarousal and re-experiencing. The analyses lend nuance and support the reviewer’s hypothesis.

As for effect size, straightforward Cohen’s d between the trigger condition and control condition at Day 2 show a small to medium effect size (d=.33). A similar effect size is found at Day 14 for the Alt.PCL scores (d=.32). We have now revised both the Results and the Discussion and suggest that participants tended to think more about the triggering passage than the control passage in the days that followed. However, we do interpret this in light of the very low overall scores in either condition. For the most part, participants did not find themselves thinking frequently about the passage they had read. We now refer to this as a statistical trend in reporting the Results on Page 18 and describe the effect relative to the effect size in the Discussion on page 25.

The cluster analysis did reveal some further depth. We created 3 clusters (Reexperiencing, Avoidance, and Hyperarousal) and looked at ALT.PCL subscores by condition and by trauma type. As for Condition, the overall statistical trend is likely driven by the hyperarousal cluster as this was the only one of the three clusters that was significant. We now discuss this in the manuscript. The overall ALT.PCL effect for trauma type was mirrored in that all three clusters also showed a significant main effect for trauma type. Post hoc analysis indicated this was due the trigger trauma group reporting more symptoms in comparison to both the other trauma and no trauma group. This is also detailed on page 18.

We both appreciate and agree with the reviewer’s comments about the inaccurate and confusing language with regard to our conclusion. When we stated that “triggering did not occur as a function of being exposed to the passage” we were referring to the PCL scores and the triggering of their own trauma. However, those with a trigger trauma did seem to think about (re-experience the passage, have more trouble sleeping) the triggering passage more than those without the triggering trauma. We have made this important distinction in the Discussion.

**Critique:** Some of the language around the conclusions/implications should be tempered, including the statement that most students “were not triggered regarding their own traumas” since it this was not directly assessed, but inferred via distress scores. It is possible that students had adverse reactions to being presented with and reading the passage that were not captured by the assessments provided here.

**Response:**

We have tempered our language and now write the following on page 30: “Those with trigger traumas did trend towards thinking slightly more about the reading as compared to those without, but most students were distressed only briefly and the triggering reading did not seem to increase symptoms regarding their own personal traumas.”

We do feel the fact that those with triggering traumas did not have higher PCL scores at Time 2 as compared to Time 1 does support our revised statement that “the triggering reading did not seem to increase symptoms regarding their own personal traumas.”

**Critique:** Minor: Typo pg 4 “without trigger warnin They”. No reference here.

**Response:**

Corrected. Thank you.

Reviewer #1:

**Critique**: Typo: “ warnin”

**Response**:

Corrected. Thank you.

**Critique:** You should mention other research studies that have analyzed trigger warnings in other academic settings as they are beginning to be reviewed in the context of medical education (ie doi: 10.1080/10401334.2017.1330690)

**Response:**

Thank you for pointing us in the right direction and pointing out this shortcoming. Since the submission of this paper, there have been other papers on this topic, so our introduction is now far more complete with six additional studies including the work by Beverly and colleagues regarding medical training.

**Critique:** Can you clarify the nature/type/size of the various universities? How did you pick these schools? Are there enrollment demographic similarities in these universities?

**Response:** The schools ranged from small private residential liberal arts schools (Middlebury College, enrollment of 2,500); Bucknell University 3,600) to a large public, commuter university (Cal Poly, San Luis Obispo, enrollment of 20,000; Coastal Carolina University, 9,000). These schools were essentially a sample of convenience in which colleagues who were willing to join the project and run the protocol did so. However, colleagues at Coastal Carolina and Cal Poly were sought because of their large enrollment and diversity of race and SES. As a basis of comparison to the figures below, 31% of students are on Pell grants nationally*. 39% are BIPOC^#^.

Gender identification, ethnicity, and a measure of SES are shown below:

Female	BIPOC	Pell grant	Percent admitted

Cal Poly	48%	43%	14%	30%

Coastal	55%	32%	37%	70%

Bucknell	51%	23%	8%	33%

Middlebury	54%	35%	13%	17%

*(<https://research.collegeboard.org/trends/student-aid/figures-tables/pell-grants-recipients-maximum-pell-and-average-pell>)

^#^https://www.aacu.org/aacu-news/newsletter/2019/march/facts-figures

We are not entirely sure what other “enrollment characteristics” the reviewer might be interested in knowing. We have included admissions rates as well.

In summary, the schools included in this would represent schools that, on average, somewhat less diverse, somewhat wealthier, and more selective than would be the national average.

While the reviewer did not make the request, we have included a Table as well as additional information in the Method on page 8.

**Critique**: The control group is small. It would have been better to incorporate a larger number of students total and had a larger control.

**Response:**

We agree with this critique and now reference it in the limitations on page 27. It would be better to have a larger control group. Our rationale for the smaller group was as follows: Because trauma and PTSD would only occur in subset of the sample, and we were interested *almost entirely* in how individuals with trauma and PTSD responded to a triggering passage, we wanted as many in that condition as possible. We really were not interested in how individuals might respond to a neutral passage. We only included a few controls as a manipulation check; primarily to confirm that our triggering passage is indeed somewhat upsetting, at least relative to a passage without an assault in it. We feel that design adequately served this purpose and allowed most students to be assigned to the triggering condition.

**Critique:** Did you ask about prior knowledge with the text? Could this have impacted your results?

**Response:**

No we did not. We now mention this as a limitation in our study on page 28. This text is a popular text frequently assigned in both high school and college. It is possible that previous exposure may have exacerbated or moderated a given individual’s response. This is probably not an issue in the trigger vs. control condition as participants were randomly assigned and therefore previous exposure to the book should have been randomly distributed among the two conditions. However, we don’t know whether previous exposure to the book differed between those with and without trigger traumas or with and without PTSD.

**Critique**: Why did you choose to end your study after 14 days? It would have been useful to monitor the impact of the triggering trauma for a longer period of time. A time point at 7 days would have helped validate your conclusions on longevity of impact.

**Response**: Fourteen days was chosen for both theoretical and practical reasons, and we were aware of the limitations that would result. However, by having a shorter study, we knew we would be able to enroll far more participants across multiple sites. With a two week window for the study, students could enroll with as few as two weeks left in the semester. We felt that the value gained by having these additional participants was greater than the value gained by assessing students for a longer period of time. We also felt that the shorter time frame would increase compliance from participants across the three assessments. While we could have assessed participants at later time points, there was little reason to believe that responses would change considerably between two weeks and four weeks. Symptoms are likely to have dissipated after two weeks and remain so thereafter.

**Critique**: How did you ensure that students did not experience a traumatic event after the experiment began between time points?

**Response:**

We were not able to ensure this. Because this was a study run with Introductory Psychology students, we worked very hard to keep the study within a one-hour time frame. There were many questions we would have liked to ask but had to sacrifice due the logistics of the study. The Day 14 assessment was really quite brief as it assessed just PTSD symptoms and distress, therefore answering the core questions of the study. An additional trauma questionnaire on Day 14 would have made the study too long although we agree it would have been valuable information; participants who had been traumatized in the intervening period would have not be included in the analyses as this would be a significant confound. However, we are fairly certain that this would not have had an effect on the study findings as the base rate of trauma in the two week period is likely to be really very low and fairly randomly distributed among the study groups.
